# Complete genome sequence of *Levilactobacillus brevis* strain DX3004 isolated from Korean fermented food

**DOI:** 10.1128/mra.00525-26

**Published:** 2026-07-21

**Authors:** Dowon Park, Bonggyu Min, Suwon Lee, Kyuchan Kwon, Chongyoon Lim, Do-Young Park

**Affiliations:** 1 Microbiome Research Institute, Dx&Vx Co., Ltd., Seoul, Republic of Korea; Nanchang University, Nanchang, Jiangxi, China

**Keywords:** *Levilactobacillus brevis*, complete genome sequence, PacBio SMRT sequencing, probiotic, antimicrobial resistance

## Abstract

We report the complete genome sequence of *Levilactobacillus brevis* DX3004. The genome consists of a single circular chromosome of 2,480,737 bp with a guanine or cytosine content of 46.0% and an average sequencing depth of 246×. Genome annotation identified 2,363 protein-coding genes, and no acquired antimicrobial resistance genes were detected.

## ANNOUNCEMENT

*Levilactobacillus brevis* is a lactic acid bacterium commonly isolated from fermented foods and widely recognized for its probiotic potential (1). This species is known for its ability to produce bioactive metabolites, including γ-aminobutyric acid (GABA), exopolysaccharides, and organic acids, which may contribute to its probiotic properties (2–4).

Here, we report the complete genome sequence of *L. brevis* DX3004 isolated from Korean fermented food kimchi. Kimchi samples were purchased from a local retail market in Guro-gu, Seoul, Republic of Korea (37.4954°N, 126.8874°E). Samples were serially diluted in sterile saline and spread onto de Man-Rogosa-Sharpe (MRS) agar plates. After incubation at 37°C for 48 h under anaerobic conditions, a single colony was selected and purified by repeated streaking. The isolate was cultured in MRS broth at 37°C for 18 h under anaerobic conditions. Genomic DNA was extracted using the Genomic DNA Purification Kit (Thermo Fisher Scientific, USA), and DNA quality was assessed using a NanoDrop 2000 spectrophotometer (Thermo Fisher Scientific, USA). Taxonomic identification was initially performed by 16S rRNA gene sequencing and subsequently confirmed by whole-genome average nucleotide identity (ANI) analysis. The closest genome in the National Center for Biotechnology Information (NCBI) Assembly database was *Levilactobacillus brevis* assembly GCA_051890795.1, with an ANI value of 99.07%, confirming the assignment of strain DX3004 to *L. brevis*.

Genomic DNA was sequenced using the PacBio Sequel IIe platform (Pacific Biosciences) and the Illumina NovaSeq X platform (Illumina). *De novo* assembly was performed using the Microbial Genome Analysis workflow implemented in SMRT Link (v25.1.0.257715), followed by polishing with Pilon (v1.22) (5–7). Unless otherwise specified, software was used according to the workflows provided by the sequencing service provider.

For PacBio sequencing, high-molecular weight genomic DNA was sheared using a Megaruptor 3 (Diagenode, Belgium), purified, and size-selected using AMPure PB magnetic beads (Pacific Biosciences). A SMRTbell library was then prepared using the PacBio SMRTbell Prep Kit 3.0 (Pacific Biosciences). Sequencing was performed on the PacBio Sequel IIe platform using the Sequel II Binding Kit 3.2 and Sequel Sequencing Kit 3.0. HiFi reads were generated using the circular consensus sequencing workflow implemented in SMRTLink (v25.1.0.257715), which performs standard-quality filtering and consensus generation. The Microbial Genome Analysis workflow implemented in SMRT Link was run using default parameters unless otherwise specified. A total of 80,718 HiFi reads (average read length, 7,561 bp; N50, 8,707 bp) were used for *de novo* genome assembly with the Microbial Genome Analysis workflow implemented in SMRT Link (v25.1.0.257715), which automatically circularized the chromosome and rotated the contig start site to the predicted origin of replication.

For Illumina sequencing, genomic DNA was fragmented using adaptive focused acoustics (Covaris), and libraries were prepared using the TruSeq DNA PCR-Free Sample Preparation Kit (Illumina). Paired-end sequencing (2 × 151 bp) was performed on the Illumina NovaSeq X platform, generating 22,924,672 reads (3.46 Gbp), of which ≥95.0% of bases had a Phred quality score ≥30. Raw Illumina reads were quality filtered to retain reads in which ≥90% of bases had a Phred quality score ≥30. Adapter removal and quality trimming were performed using Trimmomatic (v0.38) (8) with the following parameters: ILLUMINACLIP: Adapter.fasta:2:30:10:8:true, LEADING: 15, TRAILING: 15, SLIDINGWINDOW: 4:15, and MINLEN: 36. Illumina paired-end reads were aligned to the assembled chromosome and used for three rounds of error correction with Pilon (v1.22), resulting in the final polished genome assembly. The final assembly consisted of a single circular chromosome of 2,480,737 bp with a guanine or cytosine (GC) content of 46.0% and an average sequencing depth of approximately 246× (Table 1). No plasmids were detected in the final assembly. The NCBI Prokaryotic Genome Annotation Pipeline (v6.11) identified 2,363 protein-coding sequences, 15 rRNA genes, and 68 tRNA genes.

**TABLE 1:** Summary of assembly and annotation statistics for *L. brevis* DX3004

Genetic element	Length (bp)	GC (%)	No. of coding sequences	No. of rRNAs	No. of tRNAs	Sequencing depth	GenBank accession no.
Chromosome	2,480,737	46.0	2,363	15	68	246.03	CM170062.1

Antimicrobial resistance analysis using ResFinder (v4.7.2) did not identify any acquired resistance genes in the genome of *L. brevis* DX3004 (9). Analysis using the Comprehensive Antibiotic Resistance Database (v4.0.1) identified homologs related to glycopeptide resistance (vanT) and nitroimidazole resistance (nimA). However, these homologs exhibited low amino acid identity to the reference resistance determinants (31 and 48%, respectively) and were not detected by ResFinder, suggesting that they do not represent acquired or clinically relevant antimicrobial resistance genes. In particular, the vanT-related sequence is considered a distantly related homolog rather than a functional glycopeptide resistance determinant (10–13). These results indicate that DX3004 does not harbor clinically relevant acquired antimicrobial resistance genes.

## Data Availability

The accession numbers for genome sequence and raw sequencing reads for DX3004 are as follows: GenBank: CM170062.1, BioProject: PRJNA1456772, BioSample: SAMN57422333, and SRA: SRR38232826 and SRR38232827.
